# Childhood health on a planet threatened by climate change

**DOI:** 10.1016/j.jped.2024.12.002

**Published:** 2025-01-06

**Authors:** Dirceu Solé, Paulo Augusto Moreira Camargos

**Affiliations:** a Universidade Federal de São Paulo, Escola Paulista de Medicina, Departamento de Pediatria, Disciplina de Alergia, Imunologia Clínica e Reumatologia, São Paulo, São Paulo, Brazil; b Universidade Federal de Minas Gerais, Faculdade de Medicina, Programa de Pós-graduação em Saúde da Criança e do Adolescente, Belo Horizonte, Minas Gerais, Brazil

The last decade, especially the year 2024, has imposed many challenges on the planet and human beings that used to be rare, such as heat waves, and floods in different parts of North America, South America, Europe, Asia, Africa, and Oceania,^1^ forest fires, most of them anthropogenic, often in the same countries as those mentioned above and capable of devastating significant areas of vegetation and further compromising biodiversity, food production and life.^2^^,^^3^ Added to this is the burning of fossil fuels, which leads to the worsening of air pollution with its well-known health consequences, especially among the most vulnerable age groups, namely children, the elderly, and the underprivileged.^4^

These changes were significant enough that The Lancet Countdown 2024 report indicates that the world is dangerously close to violating the target of limiting the multi-annual global average warming of the Earth's surface to 1.5°C (Paris Agreement). By 2023, the average increase was 1.45°C above the pre-industrial baseline, and new highs were recorded throughout 2024.^5^

Climate extremes take lives and livelihoods. The Lancet Countdown, drawing on the expertise of 122 leading researchers from United Nations agencies and academic institutions, reveals the findings of the most concern from the collaboration's eight years of monitoring.^5^

Heat-related mortality among people over 65 years has increased by 102%, above the 65% that would have been expected without the temperature rise, as well as impairments in physical activity and sleep quality, and consequently in physical and mental health. In 2023, exposure to excessive heat put people engaged in outdoor physical activity at risk of heat stress for 27.7% more hours than the average in the 1990s and 6% more hours of sleep lost in 2023 than the average between 1986 and 2005.^5^

Since 1961, there has been an increase in the number of days of extreme rainfall across 61% of the global land area, which has increased the risk of flooding, the spread of infectious diseases, and water contamination. In parallel, 48% of the same area was affected by at least one month of extreme drought in 2023, the second-highest rate since 1951. The increase in drought events and heatwaves means that more than 151 million people will be suffering from moderate or severe food insecurity in 124 countries assessed in 2022, the highest number on record, in addition to increasing exposure to dust storms.^5^

Weather extremes and their health impacts also affect labor productivity, with heat exposure accounting for a record loss of 512 billion potential working hours in 2023, at an estimated cost in lost income equivalent to US$835 billion. Countries with low and medium Human Development Indexes were the most affected by these losses, which amounted to 7.6% and 4.4% of their Gross Domestic Product, respectively.^5^

Changes in rainfall patterns and rising temperatures favor the transmission of lethal infectious diseases, such as dengue, malaria, diseases related to the West Nile virus and vibriosis, putting populations at risk of transmission in previously unaffected areas.^5^

Dependence on fossil fuels is increasingly threatening national economies. Replacing coal-based energy with clean energy will entail an additional cumulative cost of US$164.5 billion between 2025 and 2034, which reinforces the delay in the adoption of clean and renewable energy by the majority of disadvantaged countries, which remain exposed to the damage caused by the lack of this type of energy.

Governments, companies, and transnational corporations have clearly and rapidly aggravated environmental risks. Fueled by record profits, oil and gas giants have expanded their production plans and, as of March 2024, were on track to exceed their emissions compatible with a 1.5°C increase in average temperature by 189% by 2040, 16% higher than in 2023.

Moreover, as energy prices have soared and countries’ energy systems remain dependent on fossil fuels in 2022, governments have allocated a record $1.4 trillion in subsidies to them, dwarfing any commitments to support climate actions agreed at the 28th Conference of the Parties (COP28).

The immediate engagement of citizens, companies, scientists, and international organizations concerned with climate and health is urgent and indispensable, as it helps to nurture hope that a healthy and prosperous future may still be within humanity's reach.

Echoing this panorama and with the collaboration of renowned scholars, this Supplement of *Jornal de Pediatria* is dedicated to the analysis of these topics.

The articles by Bustamante^6^ and Chong-Neto and Rosário^7^ discuss the effects of air pollution and climate change on the health of Brazilian children and adolescents. While Veras and Saldiva^8^ discuss this phenomenon in maternal, fetal, and postnatal health, Solé and Urrutia^9^ analyze respiratory health in childhood in this context and Silva et al.^10^ warn of these consequences in pediatric oncology. Neurological impairment is brought up in the texts by Nunes and da Cunha^11^ and Lopes.^12^ In addition to climate change and its consequences on biodiversity, pesticides are also part of the discussion in the article by Rodrigues et al.,^13^ microplastics in the article by Urrutia et al.,^14^ heat waves in the article by de Paula Corrêa^15^ and forest fires in the article by Rizzo and Rizzo.^16^

The indisputable evidence is: there is no room for climate denial! However, to avoid a catastrophic increase in the number of deaths, diseases and destruction of forests, urgent and decisive measures are mandatory. Now!

Be aware and responsible. Sick earth, sick children. Not just them.

## Conflicts of interest

The authors declare no conflicts of interest.
